# Our New York Quarantine Station

**Published:** 1900-01

**Authors:** 


### OUR NEW YORK QUARANTINE STATION.

THE serious charges of Mr. T. S. Cooper as to the lack of proper observance of quarantine regulations and the inefficiency of the buildings, etc., set apart for this work by the Department of Agriculture at Garfield, N. J., resulted in an investigation of the same, followed by the dismissal of Dr. W. B. E. Miller, the veterinarian in charge. We do not propose to sit in judgment as to the justness or wisdom of this decree, but if a half is true of what Mr. Cooper charges the dismissal of the inspector will only be a partial rectification of the evils to be corrected. The Government is unbending in its decree as to the ninety-days' stay as to cattle and other animals, but it only half performs its duty to the interests involved when it fails to vouchsafe to those who employ their money in importation of foreign bred stock, every care, protection, and faithful stewardship of the animals intrusted to its keeping. Let the light in, and let there be a radical correction of the evils complained of.

